# Determination of atomic vacancies in InAs/GaSb strained-layer superlattices by atomic strain

**DOI:** 10.1107/S2052252517016219

**Published:** 2018-01-01

**Authors:** Honggyu Kim, Yifei Meng, Ji-Hwan Kwon, Jean-Luc Rouviére, Jian Min Zuo

**Affiliations:** a Department of Materials Science and Engineering, University of Illinois, Urbana, IL 61801, USA; b Frederick Seitz Materials Research Laboratory, University of Illinois, Urbana, IL 61801, USA; c CEA/INAC/SP2M/LEMMA, 19 rue des Martyrs, Grenoble 38054, France

**Keywords:** atomic vacancies, defects, properties of solids, strained-layer superlattices, compound semiconductors

## Abstract

Determining vacancy in complex crystals or nanostructures represents an outstanding crystallographic problem that has a large impact on technology, especially for semiconductors, where vacancies introduce defect levels and modify the electronic structure. However, vacancy is hard to locate and its structure is difficult to probe experimentally. Reported here are atomic vacancies in the InAs/GaSb strained-layer superlattice (SLS) determined by atomic-resolution strain mapping at picometre precision. It is shown that cation and anion vacancies in the InAs/GaSb SLS give rise to local lattice relaxations, especially the nearest atoms, which can be detected using a statistical method and confirmed by simulation. The ability to map vacancy defect-induced strain and identify its location represents significant progress in the study of vacancy defects in compound semiconductors.

## Abstract 2

Atomic vacancies in complex crystals can be determined by atomic-resolution strain mapping at picometre precision using scanning transmission electron microscopy. The method is applied to InAs/GaSb superlattices.

## 1. Introduction   

Crystal defects are often characterized based on their extended strain fields and their impact on X-ray or electron diffraction. Such an extended strain field is absent for point defects (*e.g.* impurities, interstitial atoms or vacancies) at low concentrations, which makes them much harder to detect. Point defects have previously been studied using bulk spectroscopy techniques such as positron annihilation (Tuomisto & Makkonen, 2013 ▸). Although X-ray or electron diffuse scattering can be used to study certain types of point defect, such methods have not been extended to complex crystals or semiconductor heterostructures. Recent progress in atomic-resolution imaging has demonstrated that certain types of point defect such as impurity atoms (Voyles *et al.*, 2002 ▸, Oh *et al.*, 2008 ▸), vacancies in two-dimensional graphene (Hashimoto *et al.*, 2004 ▸) or antisite defects in LiFePO_4_ (Chung *et al.*, 2008 ▸) can be detected by a large difference in image contrast. In addition, quantitative image-intensity analysis based on high-angle annular dark-field (HAADF) imaging in a scanning transmission electron microscope (STEM) has been used to identify atomic vacancies (Kim *et al.*, 2016 ▸; Feng *et al.*, 2017 ▸) or individual dopant atoms (Hwang *et al.*, 2013 ▸; Ishikawa *et al.*, 2014 ▸) in a crystal. However, such contrast is generally absent in complex crystals such as compound semiconductor heterostructures because of chemical intermixing.

Here, we report on the use of atomic-resolution strain analysis for vacancy detection in InAs/GaSb SLSs using HAADF imaging in an aberration-corrected STEM and advanced image processing. The method enables the separation of strain due to composition from that caused by defects. The strain analysis is performed on the anion and cation sublattices at a high spatial resolution using the method described by Kim, Meng, Rouviére & Zuo (2017 ▸).

## 2. Experimental   

For the application of our method, we focused on InAs/GaSb strained-layer superlattices (SLSs). The InAs/GaSb SLS studied here was grown by molecular-beam epitaxy (MBE) (IQE, Bethlehem, Pennsylvania, USA) at 480°C on a GaSb substrate with 80 periods on top of a 10 nm AlSb bottom barrier and a 500 nm GaSb buffer layer. Then, a 10 nm AlSb top barrier was deposited on top of the SLS, followed by an InAs capping layer. The thicknesses of InAs and GaSb in the SLS are 4.4 nm and 2.1 nm, respectively. This SLS is undoped and was grown for photoluminescence and absorption studies for the target cut-off wavelength of 11 µm at 77 K. The InAs/GaSb SLSs have attracted considerable interest in the use of mid-wavelength (MW) and long-wavelength (LW) infrared (IR) detection for diverse scientific, civil and military applications (Smith & Mailhiot, 1987 ▸; Chow *et al.*, 1991 ▸; Mohseni *et al.*, 2001 ▸; Haugan *et al.*, 2004 ▸). When in contact, InAs and GaSb form a broken-band alignment (type II). Type II superlattices (T2SLs) comprised of alternating InAs and GaSb layers a few nanometres thick give rise to a narrow effective energy band gap with a reduced Auger recombination rate (Youngdale *et al.*, 1994 ▸), thereby making them suitable for detecting IR radiation of various wavelengths. However, the theoretical promises of InAs/GaSb T2SLs have yet to be realized, primarily due to Shockley–Read–Hall (SRH) recombination mediated by point defects, which shortens the minority-carrier lifetime (Donetsky *et al.*, 2009 ▸, 2010 ▸; Connelly *et al.*, 2010 ▸). The microscopic origin of the short carrier lifetime is still unknown, which is a major impediment to the further improvement of T2SL technologies.

For HAADF imaging, cross-section samples were prepared by mechanical polishing, followed by Ar ion milling using liquid nitrogen to minimize the ion-induced structural damage. Atomic-resolution HAADF images for strain mapping were recorded using a probe-corrected FEI Ultimate STEM operating at 300 kV (MINATEC, Grenoble, France). The HAADF images were recorded at the highest spatial resolution and image contrast (such contrast is obtained when the electron probe is channeled by the atomic column at a distance below the sample entrance surface). The electron beam was scanned parallel to the growth direction, so that the primary direction (out-of-plane direction) for the strain measurements is not affected by the so-called ‘scan fly-back error’ (which gives rise to systematic errors in the measured strain normal to the scan direction) (Chung *et al.*, 2010 ▸; Zuo *et al.*, 2014 ▸). For analysis, experimental images were carefully selected to avoid any change in contrast due to electron beam knock-on damage. HAADF image simulation was performed with the *Zmult* simulation package based on a multislice algorithm with a pixel resolution of 13.25 pm per pixel (Zuo, 2017 ▸). This simulation utilizes the absorptive potential method for electron scattering into the HAADF detector. The thickness of the TEM sample, from which the HAADF images were recorded, was estimated to be 20.1 ± 4.2 nm using position-averaged convergent-beam electron diffraction (PACBED) (Fig. S2 in the supporting information).

## 3. Results   

### 3.1. Experimental results   

**Figure 1:**
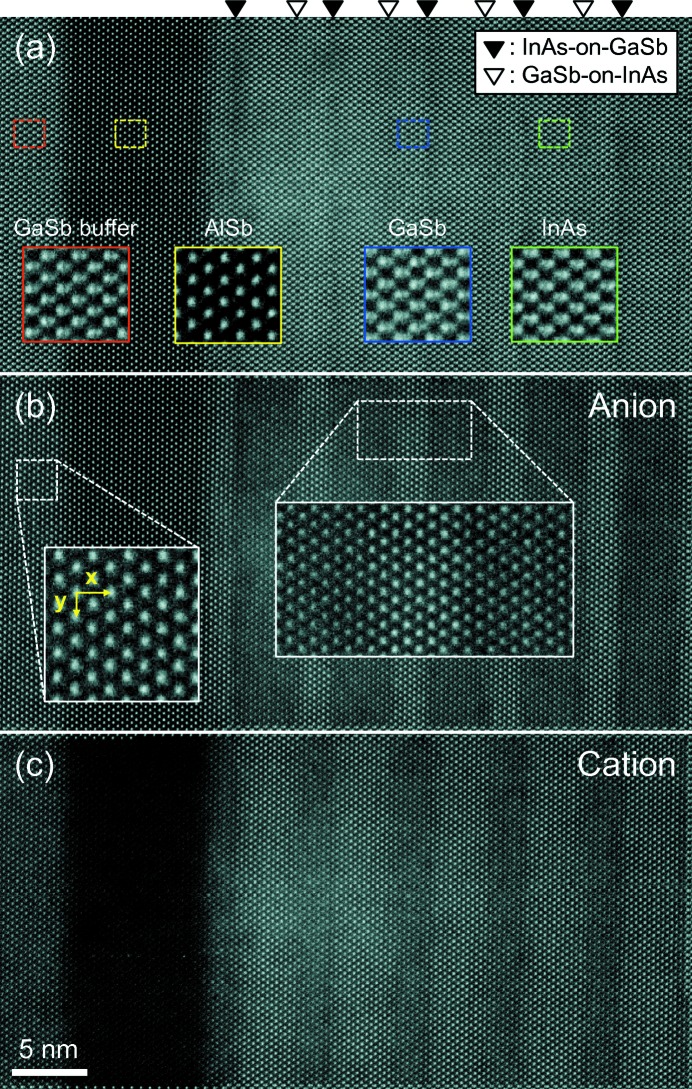
(*a*) HAADF image of InAs/GaSb T2SL along the [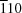] zone axis. The magnified images show, from left to right, GaSb buffer, AlSb barrier, GaSb and InAs layers. (*b*) Anion and (*c*) cation sublattice images of T2SL obtained by the peak separation method. The lattice vectors, *x* (out-of-plane) and *y* (in-plane), in the inset image in panel (*b*) are used as a reference for strain measurement. The magnified inset image of T2SL in panel (*b*) shows that the anion atomic columns are well separated from their neighboring atomic columns.

Fig. 1 ▸ displays a HAADF image of the InAs/GaSb T2SL recorded along the [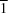
10] zone axis, including the GaSb buffer, AlSb barrier and five periods of superlattices. In this projection, atomically resolved dumbbell-like features, which consist of a pair of cation and anion atomic columns, are observed for both InAs and GaSb layers, as shown in the magnified images in Fig. 1 ▸(*a*). Since the distance between the cation and anion atomic columns is close (∼1.5 Å), the intensities of the atomic columns overlap, which makes the precise determination of the atomic column positions difficult (Peters *et al.*, 2015 ▸). To overcome this issue, we have recently developed a peak separation method (Kim, Meng, Rouviére & Zuo, 2017 ▸). The method uses Gaussian peak fitting to construct two fitted images of the cations and anions. By subtracting one of the two fitted images from the recorded HAADF image, two sublattice images are obtained with the experimental noise intact, one for anions and the other for cations (Figs. 1 ▸ *b* and 1 ▸ *c*). From these separated images, the atomic column positions can be measured and used to calculate strain using the template matching method (Zuo *et al.*, 2014 ▸). For the GaSb buffer layer, which was used as a measurement reference, we measured the standard deviations, σ_anion_ = 7 × 10^−3^ and σ_cation_ = 1 × 10^−2^, for the anion and cation lattices, respectively, corresponding to a change in distance at 2 and 3 pm, respectively, of the atomic column, representing the measurement precision (Fig. S3 in the supporting information). The difference in the σ values here arises from the different signal-to-noise ratios in the anion and cation sublattice images.

**Figure 2:**
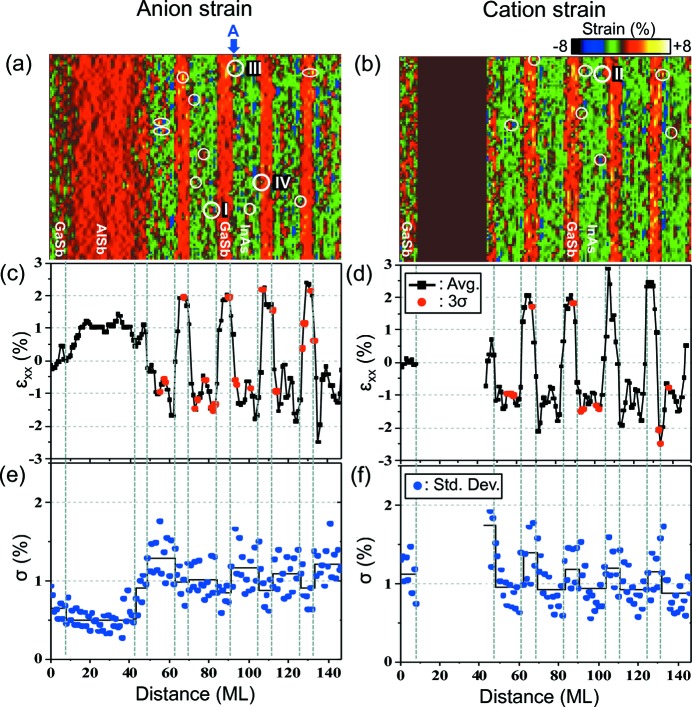
Strain maps for (*a*) anion and (*b*) cation sublattices. (*c*) and (*d*) Strain profiles averaged over 52 unit cells for the anion and cation lattices, respectively, showing the compressive strained GaSb and tensile strained InAs. (*e*) and (*f*) Standard deviations of the measured strain in each monolayer, drawn as blue circles. The solid black lines in panels (*e*) and (*f*) indicate the averaged standard deviation of the strain in each layer of the InAs/GaSb SLS. The red circles in panels (*c*) and (*d*) and the white open circles in panels (*a*) and (*b*) indicate where large strain deviations from the mean are observed. The AlSb barrier is not shown in panel (*b*) because the intensity of the Al (*Z* = 13) columns is too weak to locate its position.

Figs. 2 ▸(*a*) and 2 ▸(*b*) show measured strain ∊_*xx*_ (out-of-plane direction) maps for the anion and cation sublattices, respectively, obtained by applying the above method to a HAADF image of the InAs/GaSb T2SL recorded along the [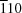] zone axis over an area of 46 × 24 nm. The measured strain is defined by 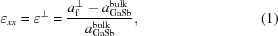
which can be related to the material strain ∊_m_

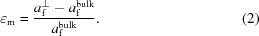


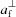
 is the local lattice parameter of the film, and 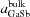 and 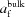 are the bulk lattice constants of GaSb and the film, respectively. See the supporting information for more details.

The HAADF image of the GaSb buffer layer, which was recorded simultaneously with the film, was used as the reference lattice to calibrate the measured strain. The major features of the two strain maps are similar: they both show that the ∊_*xx*_ values inside the nominal GaSb layers in the SLS are positive, with the maximum strain exceeding 2%, which is attributed to In incorporation into the GaSb layer (Meng *et al.*, 2014 ▸). The average strain in the nominal InAs is −1.06%. At interfaces, the strain ranges from −1.5 to −2.5%. This is more negative than for stoichiometric InAs (−1.29%) but less negative than for stoichiometric GaAs (−13.99%), showing that intermixing is present at the interface, which is in agreement with the reported compositional characterization (Kim *et al.*, 2013 ▸).

To detect point defects, we examined strain variations inside each monolayer of the T2SL. The strain distribution in each monolayer approximately follows a Gaussian distribution of width σ, and these are plotted in Figs. 2 ▸(*e*) and 2 ▸(*f*). Next, we searched for strain values lying further than 3σ from the mean (Fig. S4 in the supporting information). The locations of large strain deviations are determined and marked by white circles in Figs. 2 ▸(*a*) and 2 ▸(*b*). Within the 46 × 24 nm area of the T2SL examined, 12 are identified on the anion lattice and eight on the cation lattice. Among these, three in the anion and cation lattices are located close to each other and can be attributed to the same defect. The majority (>80%) are located near the local maxima or minima in the strain profile, marked as red dots in Figs. 2 ▸(*c*) and 2 ▸(*d*). We selected four locations for further examination,marked I, II, III and IV in Figs. 2 ▸(*a*) and 2 ▸(*b*). Here, the emphasis is placed on region I located inside the nominal InAs layer.

**Figure 3:**
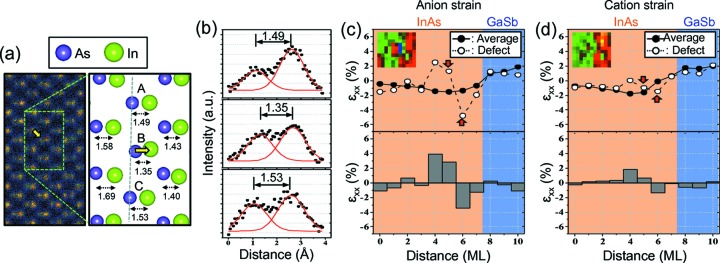
Defect atomic structure and strain distribution. (*a*) Magnified HAADF image and atomic model from location I in Fig. 2 ▸. A single atomic column, marked by the yellow arrow, shows a large displacement with respect to the averaged position of the monolayer indicated by the dotted line. The atomic distances of dumbbells labeled in the model change gradually along the growth direction due to chemical intermixing near the interface. (*b*) Gaussian peak fitting of three dumbbells (A, B and C), showing the measured atomic distances. (*c*) and (*d*) Strain profiles from the anion and cation lattices, respectively, across dumbbell B compared with the average strain profile; ML stands for monolayer. The difference between the two shows characteristic positive and negative strain differences associated with the defect.

Fig. 3 ▸(*a*) shows an atomic-resolution image and an atomic model drawn using the measured atomic column positions from region I in Fig. 2 ▸(*a*). Among the three dumbbells labeled A, B and C, the anion atomic column in dumbbell B (marked by a yellow arrow) is located where a large strain deviation (>3σ) is measured. The As atomic column is displaced toward the In atomic column, giving rise to a short As–In distance of 1.35 Å in dumbbell B, which is about 15 pm shorter than the As–In distances in the neighboring dumbbells, A and C (Fig. 3 ▸ *b*), while both the anion and cation atomic column intensities of dumbbell B are comparable with those of dumbbells A and C. Locations II and III were also found inside the nominal InAs layer, as is location I. Similar to location I, we observed local bond-length changes close to 15 pm at those two locations (Fig. S5 in the supporting information). Locations with large displacements were also observed inside the nominal GaSb layer. Location IV is an example where the Sb atomic column is displaced towards the neighboring Ga atomic column, resulting in a distinctly shorter bond length compared with neighboring dumbbells (Fig. S7 in the supporting information).

On average, the observed displacements beyond 3σ give rise to 3% deviations from the mean strain value in each monolayer, thus representing a change of >10 pm in the projected bond distances. The size of the defects extends to 1 nm. Thus, both the amount of strain and the size are much smaller than what would be expected for large defects, such as a misfit dislocation. The defects observed here likely involve a few atoms along the atomic column, since the depth of focus (DoF) for our observations is about 6 nm (DoF ≃ 1.772λ/α^2^, where λ = 1.97 pm at 300 keV, and α = 23.5 mrad is the semi-convergence angle of the electron probe) (Xin & Muller, 2009 ▸; Borisevich *et al.*, 2006 ▸).

### 3.2. Modeling results   

Local defects with small changes in bond distance have been predicted for point defects in compound semiconductors (Muratov *et al.*, 2001 ▸). Thus, to identify these, we modeled point defects in InAs, where several defects are found, using density functional theory (DFT) (see Fig. S8 and related discussion in the supporting information). The following types of point defect are considered: an As vacancy (V_As_), an In vacancy (V_In_), an As antisite defect located on an In site (As_In_), an In antisite defect on an As site (In_As_) and a Ga substitutional atom on an In site (Ga_In_). Density functional theory (DFT) calculations show that vacancy-type point defects induce a large displacement of their nearest-neighbor atoms by 48 and 50 pm for V_As_ and V_In_, respectively. In addition, the nearest-neighbor atoms move towards the vacancy position for both V_As_ and V_In_. In the case of antisite defects, the nearest-neighbor atoms around As_In_ and In_As_ are displaced by 3 and 12 pm, while a Ga substitutional atom displaces the nearest-neighbor atoms by 13 pm. The observed change in dumbbell distance from the above analysis is >10 pm. To induce such displacements by either antisite defects or compositional changes, most of the atoms in the relevant atomic column would have to be replaced by defects, which is energetically unfavorable. In addition, the extended defects, *i.e.* a cluster of antisite defects or substitutional atoms, should be shown by image contrast changes, which are not observable in our experiment. Therefore, vacancy-type defects are the most likely sources responsible for both large changes in dumbbell distance and inward displacements of neighboring atoms.

To investigate further the image characteristics of atomic-scale defects, we performed HAADF image simulations using the constructed models with different numbers of displaced atoms in the atomic column (Fig. S9 in the supporting information). The results show that an As atom displaced by 30 pm along the [001] direction in a column leads to a 0.66 pm shift of the atomic column position in the simulated HAADF image. As the number of displaced As atoms increases, the measured atomic column displacement in the simulated HAADF image also increases, eventually exceeding 10 pm with four displaced As atoms. We also examined the effect of depth of focus using model F in Fig. S9, in which the displaced As atoms are separated by a distance of ∼4.3 nm and the entire column contains five such displaced As atoms. This simulation gave an atomic column shift of 2.77 pm, which is similar to that of models A and B where the number of displaced atoms in the column is one or two. Since the depth of focus that was simulated was ∼6 nm, the above result is consistent with the focus effect. Overall, the image simulation study supports the idea that the observed displacement (>10 pm) of the atomic column could result from a number of vacancy-type point defects within the depth of focus.

**Figure 4:**
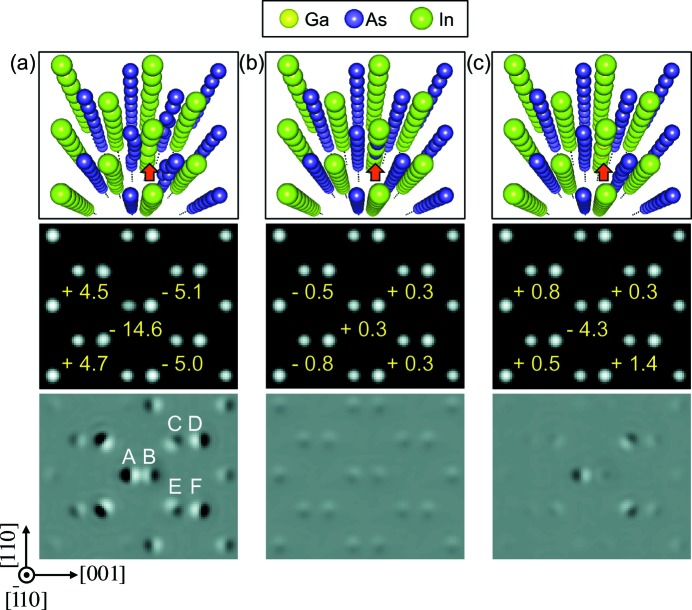
Image simulation for different types of point defect. The top row shows structure models for (*a*) vacancy, (*b*) antisite and (*c*) substitutional defects, as marked by red arrows. The middle row displays simulated *Z*-contrast images, where the atomic distances are compared with the original structure without defects. The bottom row shows difference images between structures with and without defects. The numbers in the middle row indicate the changes in bond lengths due to point defects.

Fig. 4 ▸ shows the simulated HAADF images using the relaxed structure obtained from DFT calculations for the following models: Fig. 4 ▸(*a*) two In vacancies; Fig. 4 ▸(*b*) two As_In_ antisite defects; Fig. 4 ▸(*c*) two Ga_In_ substitutions, which are all located in the In column. The two In vacancies are separated by an In atom. Four As atoms in the neighboring column A are displayed along [001], while two As atoms each in columns C and E are displaced towards the In column B. The HAADF simulation results show that the displacement of four As atoms in column A leads to a 9.9 pm displacement in the simulated HAADF image for a sample 20 nm thick, which is estimated by PACBED (Fig. S2 in the supporting information), while the In column (B) is displaced by 4.7 pm. Thus, the atomic dumbbell distance in the image is shortened by 14.6 pm, in good agreement with the experiment. Other atomic columns show much smaller displacements in the simulated image, as indicated in Fig. 4 ▸(*a*). The atomic model in Fig. 4 ▸(*b*) represents the structure with two As_In_ antisite defects in which the measured atomic column position changes very little, less than 1 pm. In the case of the two Ga_In_ substitutional atoms in Fig. 4 ▸(*c*), the measured distance of the dumbbell with Ga_In_ atoms changes by 4.3 pm. All in all, the match between the DFT models and the image simulation results suggests that the origin of the short bond lengths is vacancy-type defects, and we can also rule out antisite defects.

Lastly, we examined local strain variations near defect locations. Figs. 3 ▸(*c*) and 3 ▸(*d*) plot the strain profiles of the anion and cation lattices, respectively, across the defect at location I (open dots), before (top) and after (bottom) subtracting the average strain (solid dots, averaged over 52 unit cells along the in-plane direction). Both profiles show characteristic negative and positive differences in strain that appear as a pair, as seen in the strain map (see the cut-out strain map in Fig. 3 ▸ *c*). The extent of the strain modification is over ∼3–4 monolayers (1 nm). Compared with the simulation results obtained from the DFT models, the amount of deviation from the averaged strain measured in the cation lattice (Fig. 3 ▸ *d*) is smaller than that in the anion lattice (Fig. 3 ▸ *c*), while the two-vacancies model predicts similar strain for both cations and anions (Fig. S11 in the supporting information). The cation strain cannot be attributed to Ga substitution alone since the theory predicts almost no change in this case. Experimentally, at the location of the defect, there is 10% of Ga substitution in the In atomic column (Meng *et al.*, 2014 ▸), which amounts to two Ga atoms within the DoF. Thus, our results can be explained by the presence of ∼1–2 vacancies in the Ga-substituted In atomic column at location I.

## 4. Discussion and conclusions   

Among the observed locations (20 in total) having large atomic displacements, three occur in the Ga-rich columns inside the nominal GaSb layer. Among these, location IV shows similar strain characteristics to location I, which is likely attributed to Ga vacancies. Cation vacancies introduce deep defect levels in InAs or GaSb as they create T_2_-derived discrete energy levels just below the valence band of the bulk crystal, which act as electron acceptors (Shen *et al.*, 1992 ▸). However, in an InAs/GaSb superlattice, the valence-band edge of the effective bandgap is lower in energy than the valence-band edge of bulk GaSb due to quantum confinement effects. Defect levels created by Ga vacancies are thus in the vicinity of the effective bandgap, while In vacancies only create acceptor levels. We emphasize that our results here indicate that Ga plays an important role in defects in the InAs/GaSb superlattice depending on whether it is a vacancy formed in the Ga-substituted In atomic columns or a Ga vacancy formed in the nominal Ga atomic columns.

We note that it is important to examine whether the measured strain based on HAADF images can be representative of the bulk material. In the case of composition-modulated materials, *e.g.* superlattices, the internal stress induced by epitaxial strain is relaxed near the surface region when the specimen is thinned for (S)TEM imaging. Treacy & Gibson (1986 ▸) formulated the strain state in a superlattice using a Fourier series based on the spatial frequency *m*Λ (Λ is the periodicity of the superlattice) and Fourier components. They reported that, at the interface of a superlattice, the surface relaxation is localized to a shallow depth, thus showing the bulk behavior, while away from the interface the material tends to behave as a relaxed thin layer if *t* ≤ Λ, where *t* is the thickness of the specimen. In our previous studies, correlative analyses based on HAADF imaging, X-ray diffraction, atom probe tomography (APT) and scanning tunneling microscopy have shown good agreement on the strain and compositional information (Meng *et al.*, 2014 ▸; Kim, Meng, Klem *et al.*, 2017 ▸). Thus, it is reasonable to conclude that our present analysis based on HAADF images, obtained with the same sample preparation and imaging conditions as used in the previous study, can represent structural information on the bulk material.

To summarize, our study was made possible through a significant improvement in measurement precision to 2 pm for atomic column positions, statistical analysis of lattice strain using an aberration-corrected STEM and help from model-based simulated images using DFT. Anion and cation defects and defect locations within a superlattice consisting of layers a few nanometres thick are explicitly identified, along with the strain, at high resolution. This capability for detecting vacancies and studying their structure should stimulate further experimental and theoretical research and provide new insights for fundamental understanding of point defects in compound semiconductors and in heterostructures in general.

## 5. Related literature   

The following references are cited in the supporting information for this article: Blaha *et al.* (2001 ▸); Blöchl (1994 ▸); Freund & Suresh (2003 ▸); Vurgaftman *et al.* (2001 ▸).

## Supplementary Material

Additional tables and figures, and additional information. DOI: 10.1107/S2052252517016219/zx5011sup1.pdf
