# Supplementary material for: Determination of atomic vacancies in InAs/GaSb strained-layer superlattices by atomic strain
Source: IUCrJ. 2018 Jan 1;5(Pt 1):67–72. doi: 10.1107/S2052252517016219 (PMC5755578; doi:10.1107/S2052252517016219)
Supplement: Supplementary file 1 [file m-05-00067-sup1.pdf]

IUCrJ

Volume 5 (2018)

Supporting information for article:

Determination of atomic vacancies in InAs/GaSb strained-layer superlattices by atomic strain

Honggyu Kim, Yifei Meng, Ji-Hwan Kwon, Jean-Luc Rouviére and Jian Min Zuo

High resolution X-ray diffraction

The quality of the SLS was examined by X-ray diffraction (ω–2θ scan), which was obtained around GaSb (004) reflection using a Philips X'pert diffractometer (Pananalytical, MA). Figure S1 shows the ω–2θ scan data of the T2SL around the substrate (004) diffraction peak. All superlattice diffraction peaks between (002) and (004) are observed; only a portion of the scan is displayed.

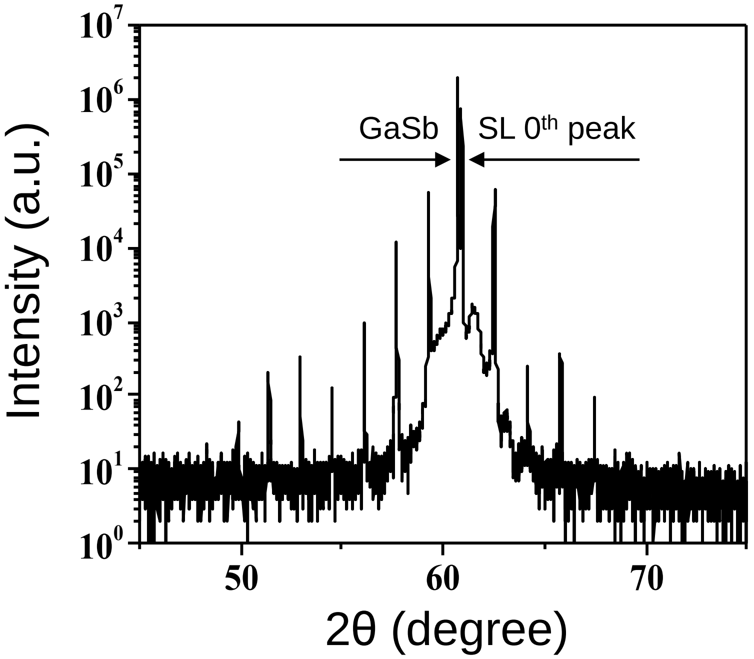

Figure S1. High-resolution x-ray ω–2θ scan data around GaSb (004) peak. The diffraction peaks show narrow full width at half maximum (FWHM), indicating a well-defined SLS at a high degree of crystallinity in agreement with the HAADF image evidence.

Thickness measurement of the TEM sample

To quantify the thickness of the TEM sample, quantitative comparisons between the experimental and simulated position averaged convergent beam electron diffraction (PACBED) patterns were carried out. Here, the mean squared error (MSE) (i.e., a sum of square intensity difference normalized to the total number of pixels) is used to evaluate the similarity between experimental and simulated PACBED patterns, given by Eq. S(1)

$$\text{MSE} = \frac{1}{N_x N_y} \sum_{i,j} [I_{exp}(x_i, y_j) - I_{sim}(x_i, y_j)]^2 \qquad \text{S(1)}$$

where $N_x N_y$ and $I(x_i, y_j)$ are the total number of pixels and pixel intensities in the PACBED pattern. Figure S2 (b) shows a plot of the MSE curve for the experimental PACBED pattern in Fig. S2 (a). By fitting this curve to the polynomial function, marked by the dotted red line in Fig. S2 (b), we estimated the thickness of the TEM sample to be 20.07 ± 4.21 nm.

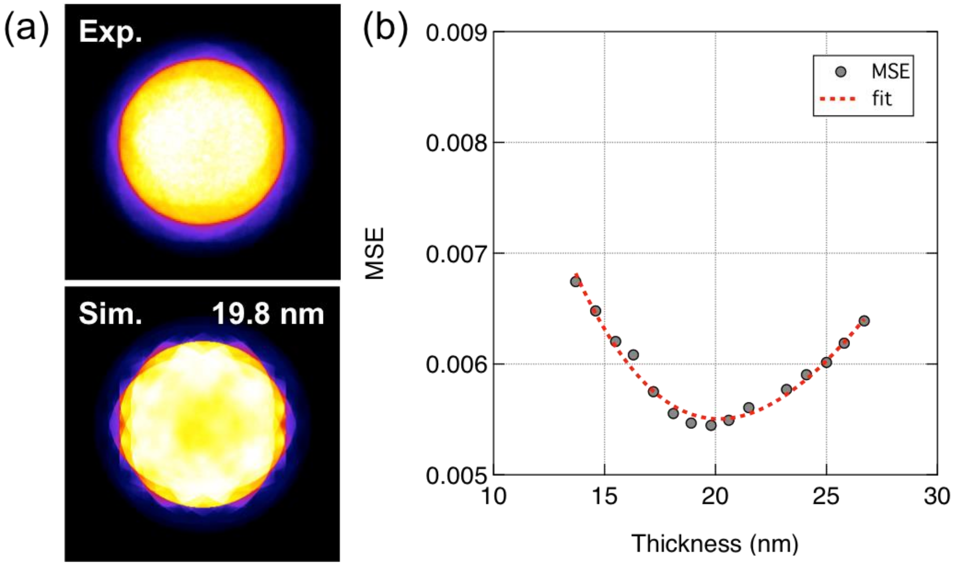

Figure S2. (a) Experimental PACBED pattern (top) recorded from the GaSb buffer layer and a simulated PACBED pattern (bottom) for a 19.8 nm thick GaSb along the [110] projection. (b) Plot of the MSE curve and the fit to a polynomial function.

Measurement precision in atomic column positions

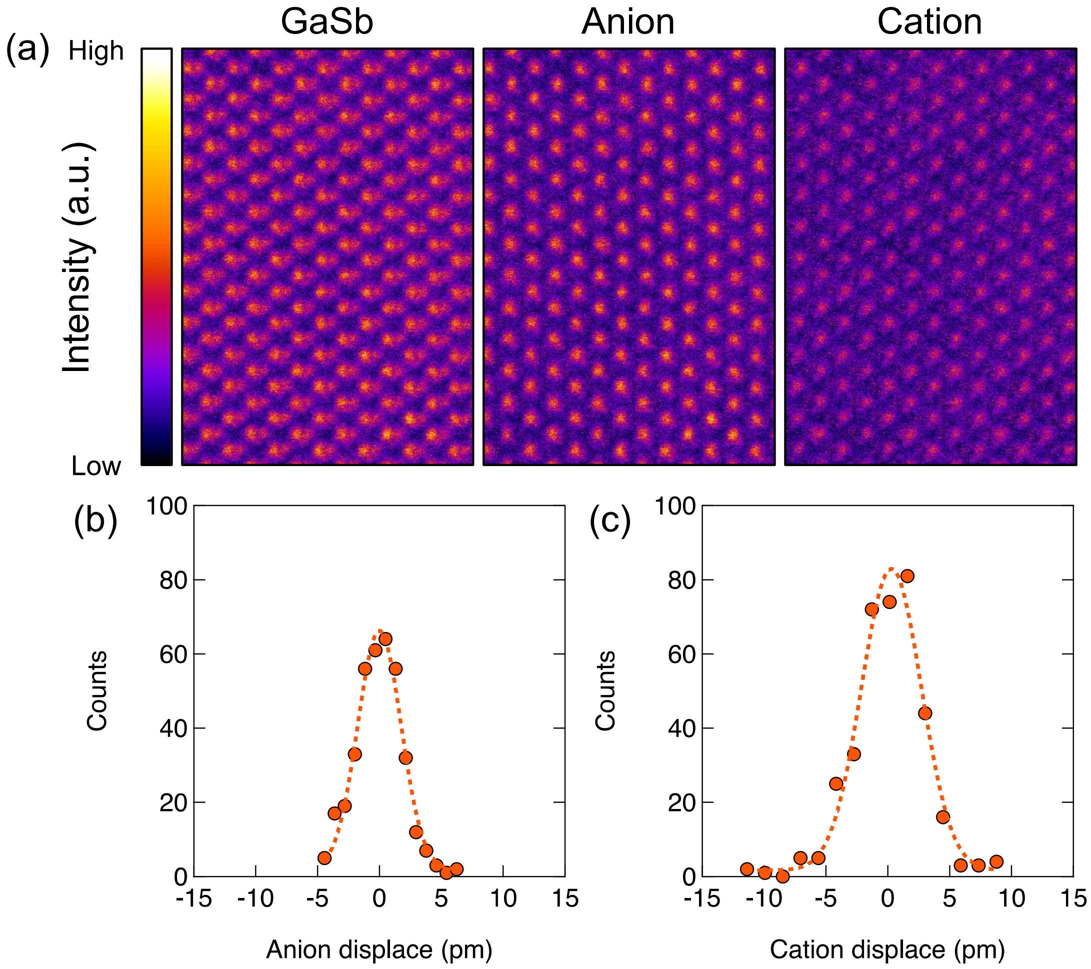

Figure S3. Precision in the measurement of atomic column positions. (a) HAADF images of a GaSb substrate and sublattice images obtained using the peak separation method. (b) and (c) The histograms of the measured displacements from the anion and cation sublattices, showing the measurement precisions along the out-of-lattice direction are 2 and 3 pm, respectively.

The measured strain and material strain

The strain ($\varepsilon^{\perp}$) in our strain map is measured based on the out-of-plane lattice mismatch between the film and the GaSb buffer layer, given by Eq. S(2)

$$\varepsilon^{\perp} = \frac{a_f^{\perp} - a_{GaSb}^{bulk}}{a_{GaSb}^{bulk}} \qquad S(2)$$

where $a_f^{\perp}$ and a_{GaSb}^{bulk} (6.0959 Å) are the out-of-plane lattice constants of the deposited film and the bulk lattice constant of GaSb, respectively. Since the T2SL is lattice-matched along in-plane direction with GaSb buffer layer, $a_f^{\perp}$ can be calculated by, (Freund & Suresh, 2003)

$$a_f^{\perp} = a_f^{bulk}\left(1 - \frac{2\nu_f}{1-\nu_f}\varepsilon_f^{\parallel}\right) \qquad S(3)$$

where $\varepsilon_f^{\parallel} = (a_f^{\parallel} - a_f^{bulk})/a_f^{bulk}$ is the in-plane strain of the film and ν_f, a_f^{bulk} are the Poisson ratio and the bulk lattice constant of the film. Using the reported parameters of bulk lattice constants and Poisson ratios(Vurgaftman *et al.*, 2001), the out-of-plane lattice mismatches can be calculated for the possible chemical bonds in InAs/GaSb T2SL.

The material strain in the superlattices differs significantly from the measured strain ($\varepsilon^{\perp}$) defined above. Introducing the misfit, $m = (a_f^{bulk} - a_{GaSb}^{bulk}) / a_{GaSb}^{bulk} = a_f^{bulk}\left(a_{GaSb}^{bulk}\right)^{-1} - 1$ between film and GaSb substrate, one obtains

$$(\varepsilon_m + 1) = a\left(a_{GaSb}^{bulk}\right)^{-1} = (\varepsilon^{\perp} + 1) / (m + 1) \qquad S(4)$$

Thus, to first order in strain, we have $\varepsilon_m \approx \varepsilon^{\perp} - m$.

Identification of defects using 3σ criterion

Identification of defects is made by analyzing strain in individual monolayers parallel to the superlattice. Figure S4 shows two examples. Each monolayer contains 104 points. The histogram of distribution is used to identify those lying outside the 3σ range.

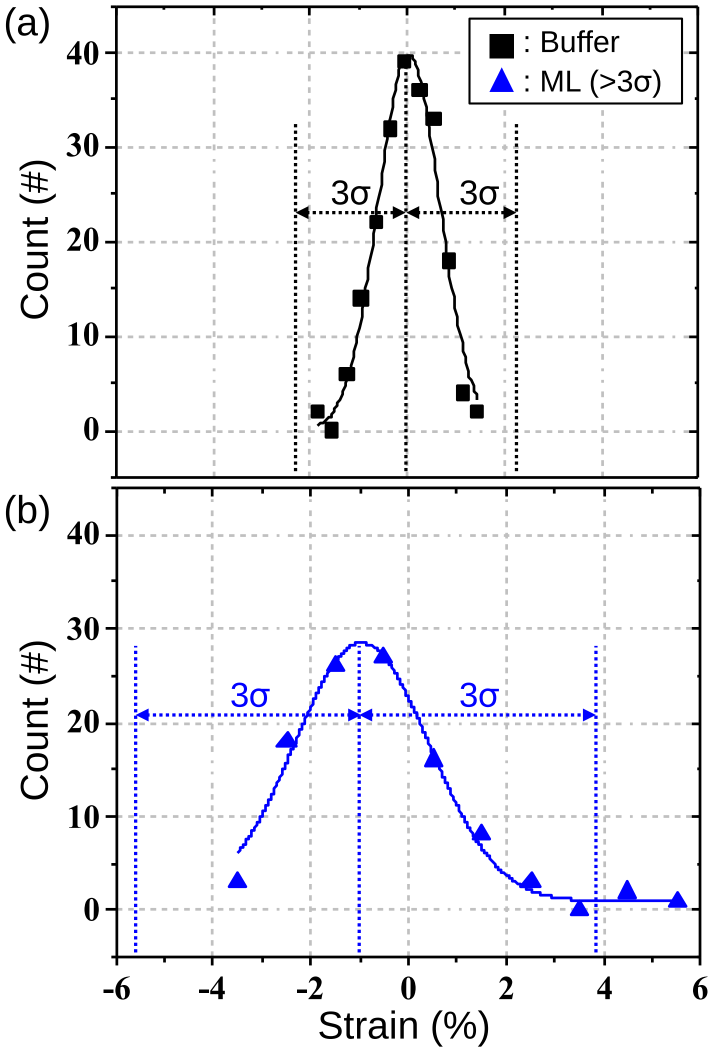

Figure S4. Strain distribution in a single monolayer in InAs/GaSb T2SL. (a) Strain distribution obtained in the GaSb buffer layer where the strain field is minimal. (b) Strain distribution from the monolayer A in Fig. 2a contains a few strain values lying outside 3σ from the mean.

Strain and atomic displacement in locations II, III and IV

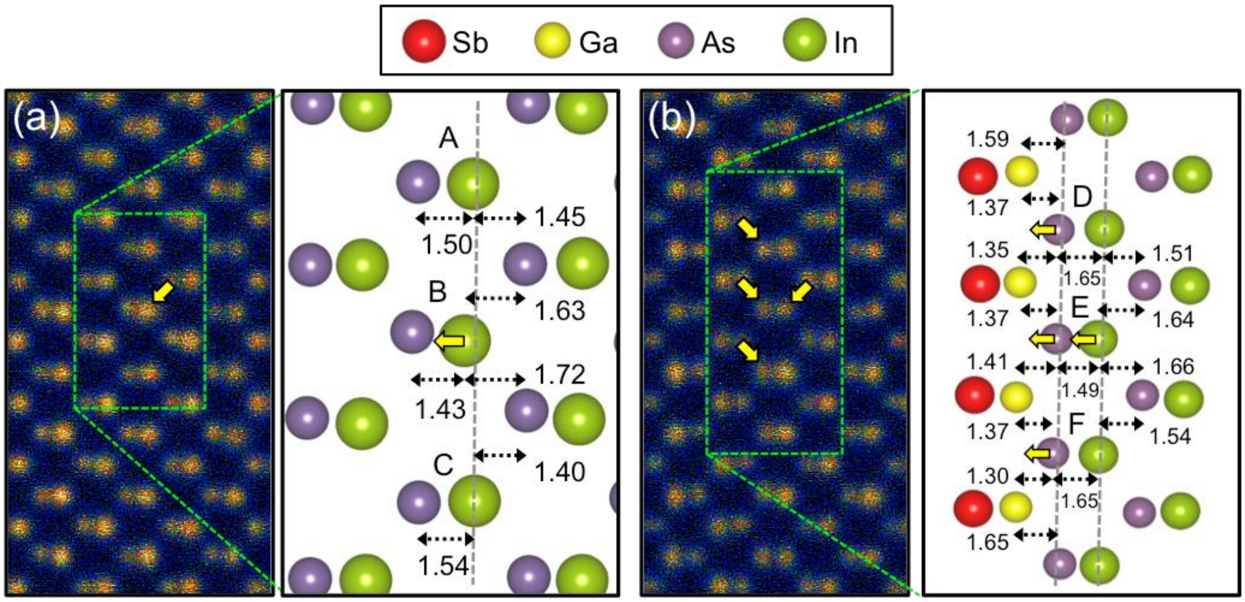

Figure S5. Atomic resolution images and atomic models from regions II and III. (a) and (b) Magnified HAADF images from the locations marked by regions II and III in Fig. 2b and a, respectively. The atomic models correspond to the green dotted box regions. The single cation atomic column, marked by the yellow arrow in (a), is detected with 'beyond 3σ criterion' while the displacements of a cluster of atomic columns are detected in (b). The yellow arrows in (b) indicate three anion and one cation columns largely displaced from the averaged position of the monolayer (grey dotted lines). The local bond lengths near the large displacement of atomic column are measured and listed in each atomic model. Note that the relatively large atomic distances in dumbbells D and F compared to that of dumbbell E are due to the GaAs-like bonds at E, as evidenced by the relative intensities of the atomic columns.

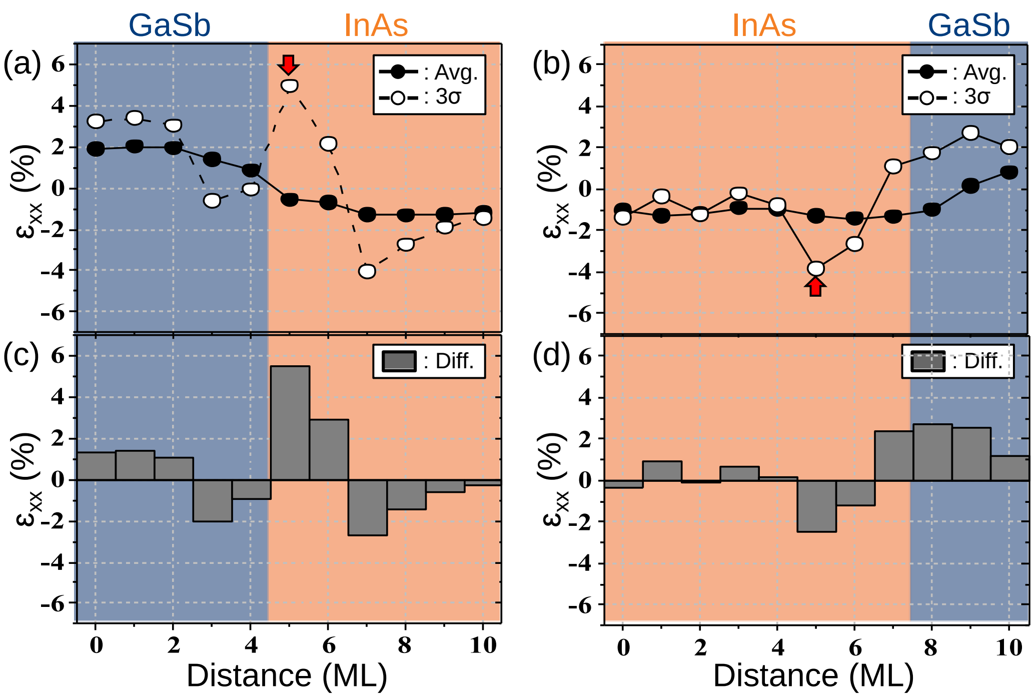

Figure S6. Strain profiles from regions II and III. (a) and (b) The averaged strain profiles (solid dots) from regions II and III are plotted with the strain profile (open dots) containing the largely displaced atomic columns, marked by yellow arrows in Fig. S5 (a) and (b). (c) and (d) The differences between those strain profiles are plotted for regions II and III. The red arrows in (a) and (b) indicate the strain values induced by the atomic columns with large displacements.

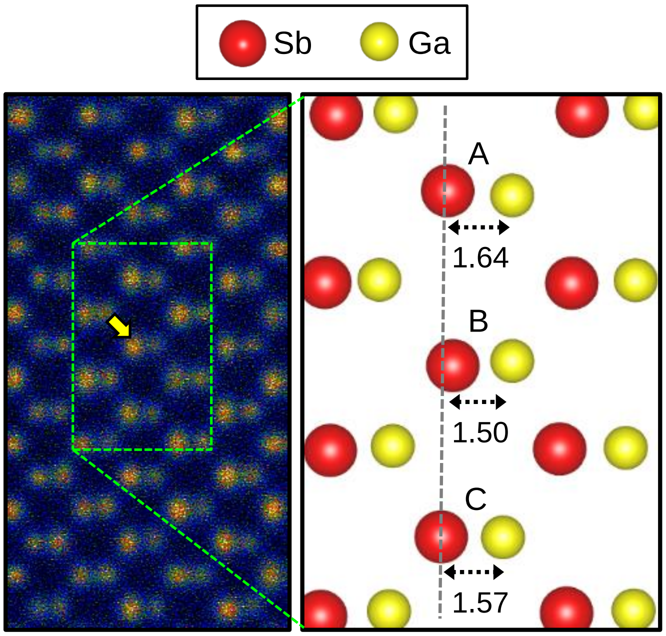

Figure S7. Magnified HAADF image and atomic model from the location marked by region IV. The single anion atomic column, marked by the yellow arrows is detected as ‘beyond 3σ criterion’. The atomic distances of three dumbbells, marked by ‘A’, ‘B’, and ‘C’, are measured at 1.64, 1.50, and 1.57 Å, respectively. The atomic distance of dumbbell ‘B’ is distinctly shorter than those of neighboring dumbbells due to the displacement of the nominal anion atomic column, which is attributed to the vacancy (or vacancies) in the nominal cation atomic column.

Modeling of point defects

The calculations are performed based on the full-potential linearized-augmented plane wave method using WIEN2k package.(Blaha *et al.*, 2001) The exchange-correlation functional is treated within the generalized gradient approximation by Perdew, Burke, amd Ernzerhof (GGA-PBE) as implemented in WIEN2k package.(Blöchl, 1994) In the present study, we made four structures involving different types of point defects in InAs: As vacancy (V_{As}); In vacancy (V_{In}); As anti-site defect located on a In site (As_{In}); and In anti-site defect located on a As site (In_{As}), as shown in Fig. S8 (b-e). The supercell [Fig. S8 (a)] with the dimension of a = 12.12 Å, b = 17.14 Å, c = 8.57 Å and 4×3×6 k-point grid were used. All atoms are free to move until the force tolerance of 2 mRy/bohr was reached. A summary of the lattice relaxation result is shown for vacancy and anti-site point defects in Tables S1 and S2, respectively. The averaged displacements of four nearest neighboring atoms induced by V_{As} and V_{In} are about 48 and 50 pm, respectively. The displacements of the second nearest neighboring atoms are reduced at < 20 pm for both vacancies. On the other hand, the anti-site defects induce the relaxation of the nearest neighboring atoms by 3 and 12 pm for As_{In} and In_{As}, respectively. Similar to the vacancies, the displacements of the second nearest neighboring atoms are significantly reduced at 0.3 and 5 pm, respectively.

The DFT result shows that one vacancy defect displaces four nearest neighboring atoms toward the vacancy position. Observed along <110> direction, two of them are located in the same dumbbell pair right next to point defects and another two are separately located in different dumbbell pairs, e.g. two As atoms in the same dumbbell pair with In vacancy and two As atoms located at right upper and lower side of In vacancy are displaced in Fig. S8 (c).

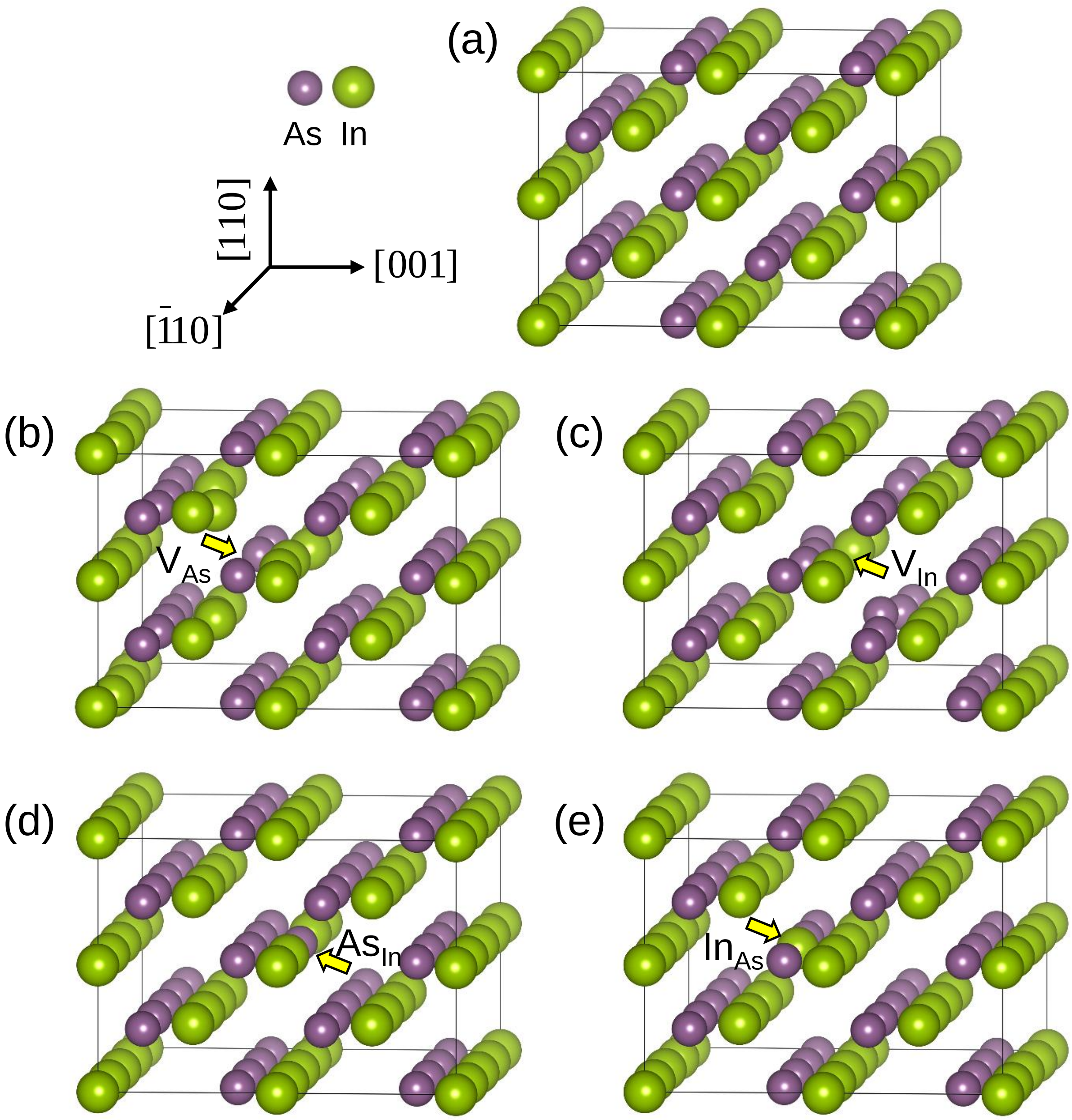

Figure S8. Structure relaxation by different types of point defects in InAs. (a) The supercell of InAs for DFT calcuations. The lattice relaxation results induced by different types of point defects such as (b) and (c) As and In vacancies and (d) and (e) As_{In} and In_{As} anti-site defects are displayed.

TABLE S1. Relaxation around the vacancy point defects, V_{As} and V_{In}. NN and 2NN represent the first nearest and second nearest neighboring atoms from the defects. Locations of all four nearest neighboring atoms are listed while the location of one second nearest neighboring atom is listed.

		V_{As}							V_{In}						
		Original (Å)			Relaxed (Å)			Disp (pm)	Original (Å)			Relaxed (Å)			Disp (pm)
Type		x	y	z	x	y	z		x	y	z	x	y	z	
NN	1	3.029	6.426	2.142	3.413	6.426	2.446	49	4.544	6.426	4.284	4.854	6.859	4.284	53
	2	3.029	6.426	6.426	3.413	6.426	6.122	49	4.544	10.710	4.284	4.854	10.278	4.284	53
	3	6.058	4.284	4.284	5.763	4.661	4.284	48	7.573	8.568	2.142	7.244	8.568	2.464	46
	4	6.058	8.568	4.284	5.762	8.191	4.284	48	7.573	8.568	6.426	7.244	8.568	6.104	46
2NN		1.515	4.284	6.426	1.634	4.405	6.382	18	3.029	6.426	6.426	3.160	6.465	6.294	19

TABLE S2. Relaxation around the antisite point defects (AD), As_{In} and In_{As}.

		As_{In}							In_{As}						
		Original (Å)			Relaxed (Å)			Disp (pm)	Original (Å)			Relaxed (Å)			Disp (pm)
Type		x	y	z	x	y	z		X	y	z	x	y	z	
AD		6.06	8.57	4.28	6.08	8.59	4.31	4	4.544	6.426	4.284	4.618	6.455	4.325	9
NN	1	4.54	6.43	4.28	4.58	6.47	4.29	6	3.029	6.426	2.142	2.967	6.424	2.096	8
	2	7.57	8.57	2.14	7.56	8.57	2.17	3	3.029	6.426	6.426	2.938	6.423	6.516	13
	3	4.54	10.71	4.28	4.55	10.70	4.28	2	6.058	4.284	4.284	6.105	4.182	4.281	11
	4	7.57	8.57	6.43	7.58	8.57	6.45	2	6.058	8.568	4.284	6.134	8.706	4.279	16
2NN		3.029	6.426	6.426	3.028	6.427	6.428	0.3	1.515	4.284	6.426	1.483	4.254	6.446	5

TABLE S3. Relaxation around the substitutional atoms (ST), Ga_{In}.

		Ga_{In}						
		Original (Å)			Relaxed (Å)			Disp (pm)
Type		x	y	z	x	y	z	
ST		6.06	8.57	4.28	6.06	8.57	4.28	0
NN	1	4.54	10.71	4.28	4.63	10.59	4.28	14
	2	4.54	6.43	4.28	4.63	6.54	4.28	14
	3	7.57	8.57	6.43	7.48	8.57	6.32	13
	4	7.57	8.57	2.14	7.48	8.57	2.24	13
2NN		9.09	10.71	6.43	9.06	10.68	6.41	4

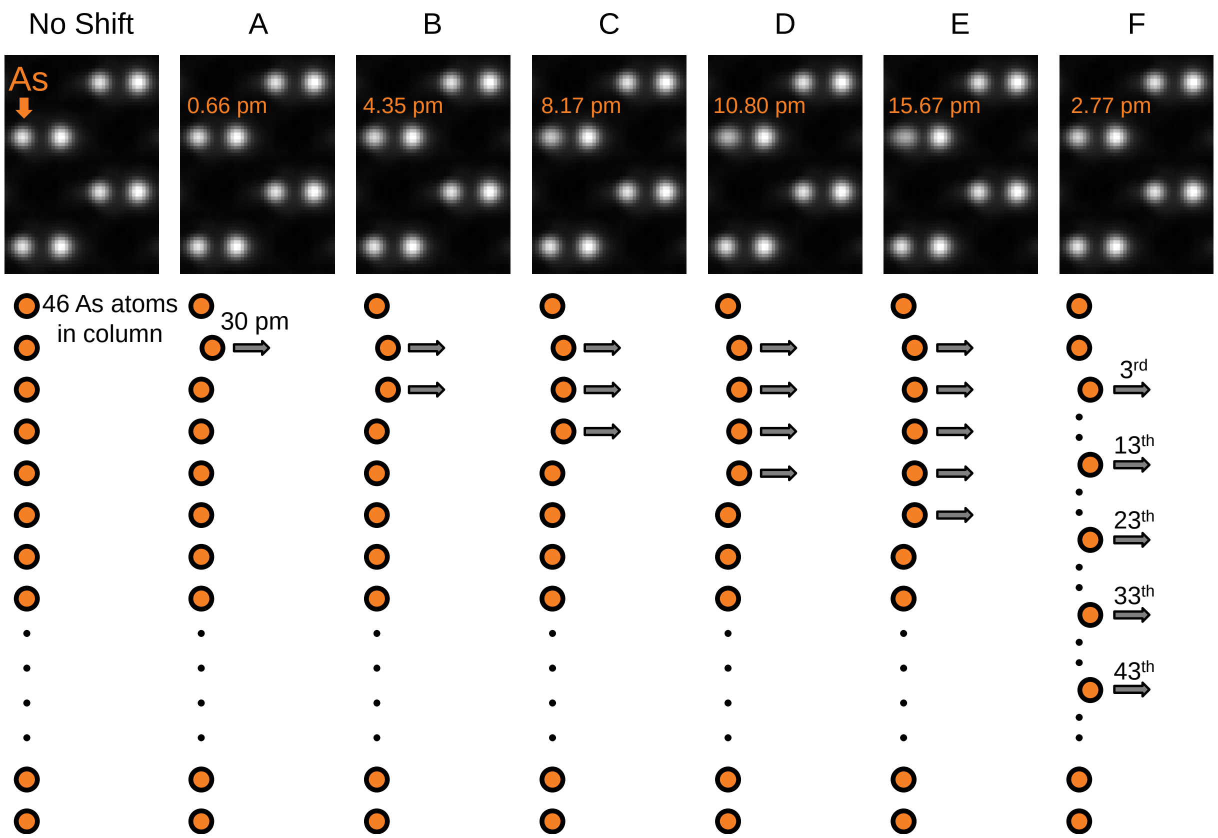

Figure S9. Simulated HAADF images of InAs along [110] zone axis. Below each model, the corresponding structure models for the As column are drawn. The As atoms in the column, indicated by the orange arrow, are intentionally displaced by 30 pm. The number of displaced As atoms ranges from one to five in the model A - E. The model F involves five displaced As atoms which are well separated from each other. The amounts of displacement of As column are listed in each simulated HAADF image.

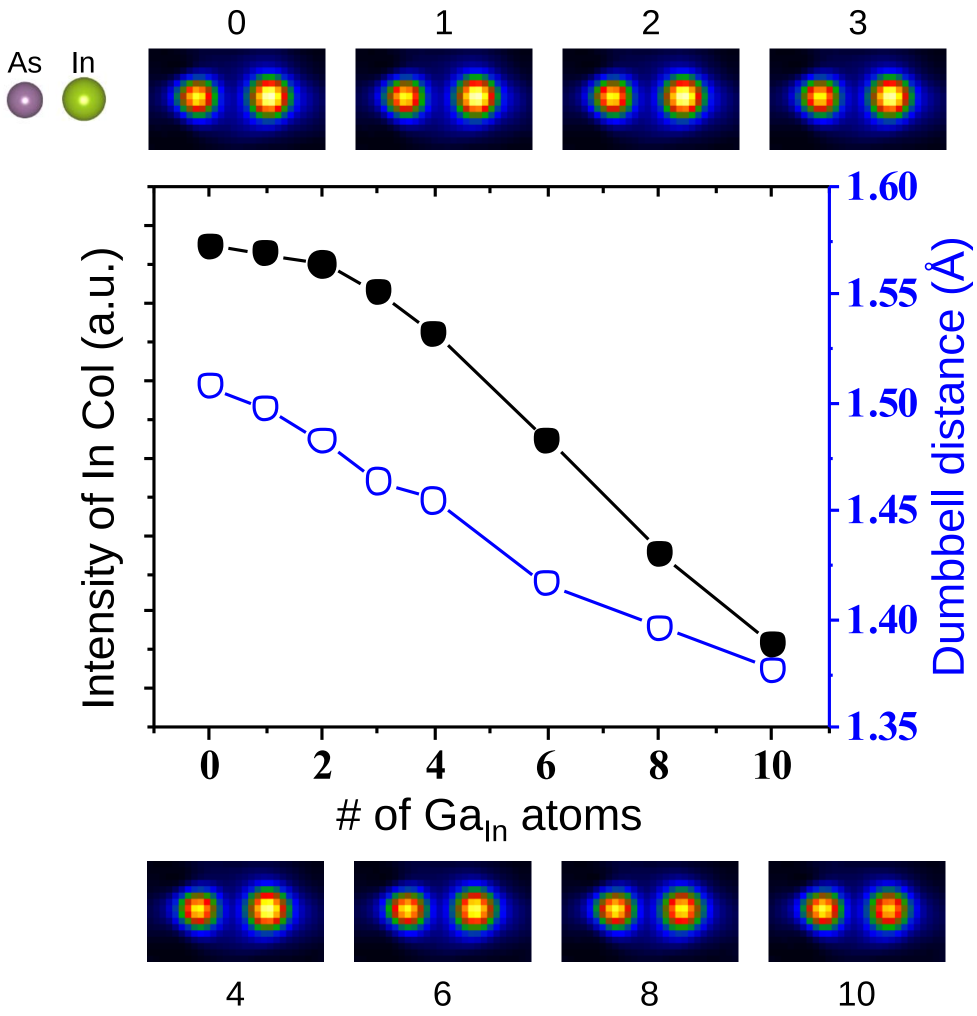

Figure S10. Simulated HAADF images of InAs with different numbers of Ga_{In} substitutional atoms. The model structure (20 nm thick) studied is InAs containing different numbers of Ga_{In}. The intensity of the nominal In column and the dumbbell distance decrease as the number of Ga_{In} increases. With eight Ga_{In} atoms, the amount of displacement of As atomic column exceeds 10 pm, which can be detected with 3σ criterion. However, the intensity of the nominal In significantly decreases at the level of As atomic column. This feature is distinctly different from the experiment observation, where the dumbbell distances change by more than 10 pm is detected with only a small contrast change. The short dumbbell distance with relatively high intensity, therefore, arises from the vacancy type point defects rather than compositional variation.

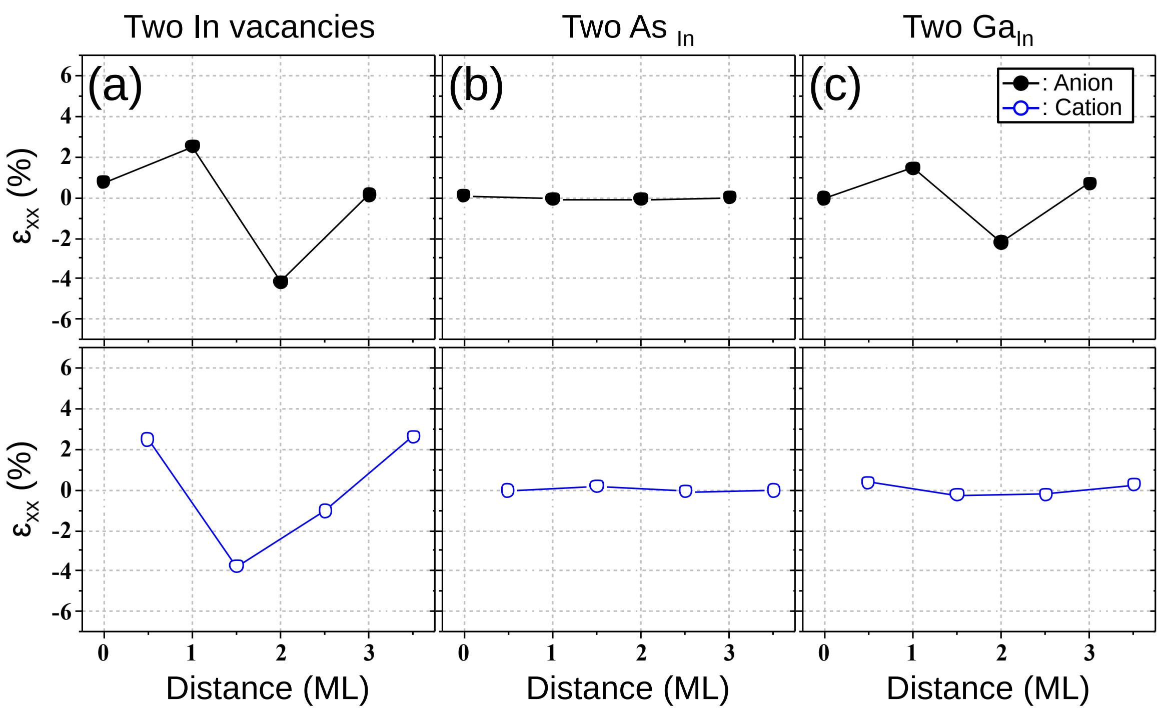

Figure S11. Strain profiles from simulated HAADF images of InAs with different types of defects. (a) Strain profiles obtained from InAs with two In vacancies. The atomic displacements induced by vacancies change local bond lengths, creating negative and positive strain differences for both anion and cation strain profiles. (b) Strain profiles obtained from InAs with two As_{In}. As anti-site defects on In sites result in negligible changes in bond length for both anion and cation lattices. (c) Strain profiles obtained from InAs with two Ga_{In}. A compositional change in cations leads to local bond length changes for the anion lattice while the bond length in cation lattice is negligible. The experimentally observed strain profile (Fig. 3 in the main text) shows the characteristic strain profiles with negative and positive strain differences for both anion and cation lattices. However, the amount of strain difference from the cation lattice is smaller than that from the anion lattice. According to previous results with XRD and atomic probe tomography (APT) (Meng *et al.*, 2014), there are a certain degree of chemical intermixing near the interface between InAs and GaSb. Thus, the strain profiles obtained near region I in Fig. 3 result from local bond length changes induced by both In vacancies and compositional intermixing.

Sample thickness effect on the measurement of atomic column positions

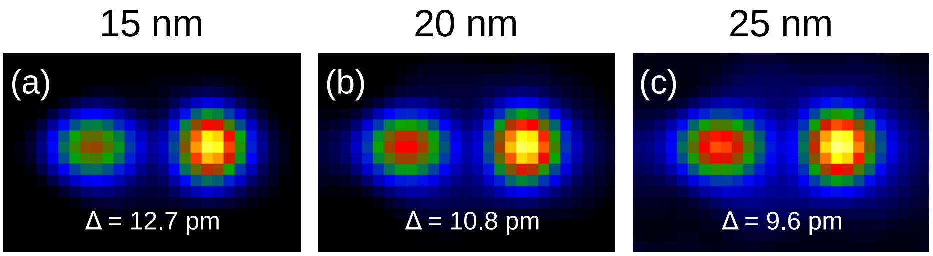

Figure S12. Simulated HAADF images with different specimen thicknesses. To verify the specimen thickness effect on measured displacement of atomic columns in HAADF images, image simulations are performed with InAs with four As atoms displaced by 30 pm toward In atomic column (model D in Fig. S9). (a) – (c) The amounts of As atomic column displacements are 12.7, 10.8, and 9.6 pm for specimen thicknesses of 15, 20, and 25 nm, respectively. On average, for each nanometer thickness variation, there is about 0.3 pm change in measured atomic column positions. This result demonstrates that the large displacement of atomic columns induced by point defects can be detectable over relatively large thickness variations.

References

Blaha, P., Schwarz, K., Madsen, G., Kvasnicka, D. & Luitz, J. (2001). *Schwarz (Vienna University of Technology, Austria, 2001).*

Blöchl, P. E. (1994). *Physical Review B* **50**, 17953-17979.

Freund, L. B. & Suresh, S. (2003). *Thin film materials: stress, defect formation and surface evolution*. Cambridge University Press.

Meng, Y., Kim, H., Rouviére, J.-L., Isheim, D., Seidman, D. N. & Zuo, J.-M. (2014). *Journal of applied physics* **116**, 013513.

Vurgaftman, I., Meyer, J. & Ram-Mohan, L. (2001). *Journal of applied physics* **89**, 5815-5875.
